# The Tropical Andes Biodiversity Hotspot: A Comprehensive Dataset for the Mira-Mataje Binational Basins

**DOI:** 10.1038/s41597-024-03463-1

**Published:** 2024-07-16

**Authors:** Mario H. Yánez-Muñoz, Pablo Jarrín-V., Jorge Brito M., Roberto Román-RR, Juranny M. Astorquiza, Aida E. Baca, Paul A. Baker, Patricia Bejarano-Muñoz, Eric Y. Cuesta R., Efraín Freire, César Garzón, Jorge Gómez-Paredes, William Klinger, Luz E. Lagos, Wilderson Medina, Patricio Mena-Valenzuela, Luis J. Mosquera R., Robinson S. Mosquera M., Yirsela Murillo, Yiscar D. Murillo A., Edsson Nagle R., Germán Narváez, Stuart Pimm, Cecilia Proaño, Francisco J. Prieto A., Zoraida Quezada, Giovanny Ramírez, Reimer Rengifo I., Luis E. Rentería M., Miguel A. Urgilés-Merchán, Lady Vargas, Carla Valdospinos, Zulmary Valolyes, Diego J. Inclán

**Affiliations:** 1 Consorcio Binacional Mira Mataje (CBMM), Quito, Ecuador; 2 Instituto Nacional de Biodiversidad, Rumipamba 341 y Av. de los Shyris, Quito, Ecuador; 3 Instituto de Investigaciones Ambientales del Pacífico, Quibdó, Colombia; 4 Universidad de Nariño, Nariño, Colombia; 5 Nicholas School of the Environment, Duke University, 27708 Durham, NC USA; 6 Beyond One Foundation (Secretariat of the UN Sustainable Development Solutions Network for the Andean Region -SDSN Andes-), Quito, Ecuador

**Keywords:** Biodiversity, Taxonomy

## Abstract

We present a flora and fauna dataset for the Mira-Mataje binational basins. This is an area shared between southwestern Colombia and northwestern Ecuador, where both the Chocó and Tropical Andes biodiversity hotspots converge. We systematized data from 120 sources in the Darwin Core Archive (DwC-A) standard and geospatial vector data format for geographic information systems (GIS) (shapefiles). Sources included natural history museums, published literature, and citizen science repositories across 13 countries. The resulting database has 33,460 records from 6,821 species, of which 540 have been recorded as endemic, and 612 as threatened. The diversity represented in the dataset is equivalent to 10% of the total plant species and 26% of the total terrestrial vertebrate species in both hotspots. The dataset can be used to estimate and compare biodiversity patterns with environmental parameters and provide value to ecosystems, ecoregions, and protected areas. The dataset is a baseline for future assessments of biodiversity in the face of environmental degradation, climate change, and accelerated extinction processes.

## Background & Summary

With less than 1% of the planet’s surface, the Tropical Andes and Chocó biodiversity hotspots shelter the richest and most endemically dense biota of flora and fauna on Earth^1^. Both, in northwestern South America, are a high priority for conservation at local, regional, and global scales. They require transnational collaborative networks to conserve their biodiversity^1–5^.

In the transboundary basins of the Mira and Mataje rivers, the tropical forests of the Chocó converge with the tropical Andes of northern Colombia and Ecuador. These watersheds are of significance to conservationists and researchers in northwestern South America. Several lineages described as new species show the importance of these basins in the mechanisms of speciation and geographic distribution of flora and fauna^5–16^. Nevertheless, there have been no documented efforts to consolidate the available information on species occurrences into a unified dataset for this important conservation region.

The armed conflict and geopolitical setting in this national border, between southern Colombia and northern Ecuador, have prevented bioexploration in recent decades^17^. Valuable remnants of natural vegetation in both basins require urgent conservation actions^9^. It is within this context that the project consortium named “Biodiversity Conservation in the Binational Basins of the Mira-Mataje Rivers (MMRB): Construction of Biophysical and Socio-environmental Bases for the Conservation and Adaptive Management of Ecosystem Services”, has the purpose of influencing and participating in decision-making. It aspires to improve the capacity of stakeholders and decision-makers to conserve ecosystems and optimize the management of its environmental services. One main component of the MMRB was to establish flora and fauna datasets of collected and observed specimens from the basins in Colombia and Ecuador. The provided datasets can be enriched with additional records through new field expeditions for exploring current sampling gaps. We encourage the use and updating of the presented datasets.

## Methods

### Study area

**Fig. 1:**
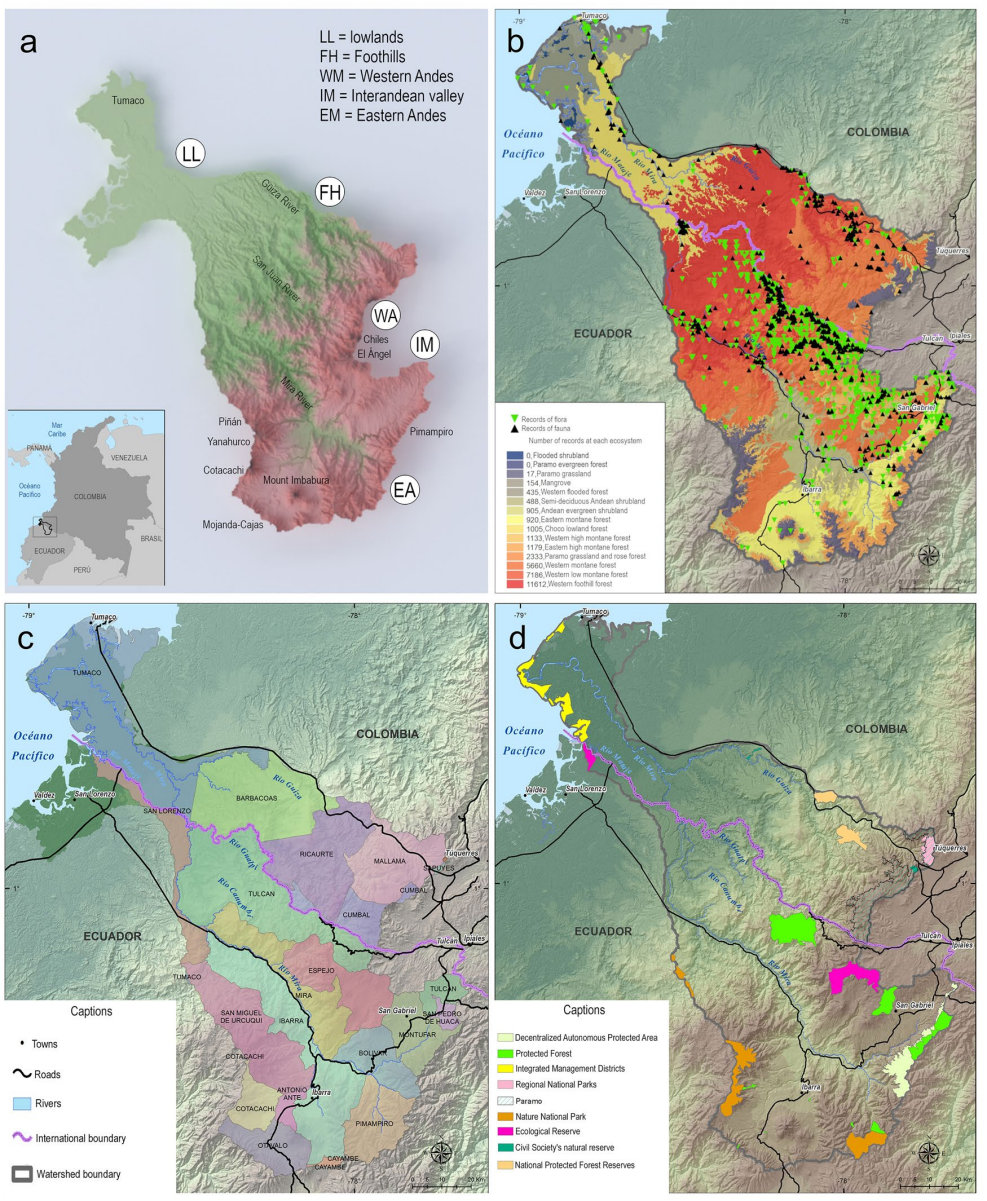
Study area: the Mira-Mataje binational basin. (**a**) Shaded relief map with contrasting colors showing a cutoff at mean elevation = 2072 m (green < 2072 m, red > 2072 m). (**b**) Georeferenced flora and fauna records that are included in the presented dataset. (**c**) Political boundaries, roads, rivers, and watershed boundaries. (**d**) Protected areas.

The Mira and Mataje binational basins are in northwestern Ecuador and southwestern Colombia; they cover an area of 1,045,965 hectares. They originate in Ecuador, in the Andean Massif of the Eastern Cordillera of the Andes in the Pimampiro region. To the north, they connect through the inter-Andean mountains of Tulcán, with the Western Andes in the El Ángel paramo. To the south, they are delimited by the Mojanda and Cajas crossroads, connecting to the west with the Cotacachi-Yanahurco-Piñan mountain system (Fig. 1). Their waters flow through moorlands, foothill cloud forests, and rainforests until reaching Cabo Manglares in Tumaco, where they empty into the Pacific Ocean in Colombia. They subdivide into six sub-basins, have more than 15 ecological systems, and their elevation gradient ranges from 0 to 4800 m.

### Data sources and data compilation

We obtained a collection of 35 data tables for records of fauna and a collection of 36 data tables for records of flora (Supplementary Tables S1 and S2). We accessed these data tables from October 2019 to October 2022. The flora collection included records of vascular plants, while the fauna collection included records of mammals, birds, reptiles, and amphibians. We obtained the data tables from various sources, including government agencies, non-governmental organizations, and academic institutions. Data was delimited geographically to those records belonging to the Mira-Mataje binational basins.

### Data organization

We systematized and reorganized the information on all the data tables to create a new dataset based on the Darwin Core Archive (DwC-A) standard^18^. The DwC-A provides a way to package biodiversity data and metadata in a self-contained and machine-readable format. The DwC-A serves for sharing biodiversity data and is primarily based on the occurrence in nature of taxa, as documented by observations, specimens, samples, and related information^18^. An accompanying shape file is also provided. The shapefile delimits all ecosystems in the Mira-Mataje binational basins, and provides a tabulated measure of total records, total recorded species, number of endemic species, and number of threatened species. Data organization was carried out by hand and by a single researcher who applied available filters to the selected source data tables and extracted the corresponding data records. Data organization followed the DataONE Best Practices as a guideline^19^

### Data cleaning

To ensure accuracy, completeness, and consistency, we did manual and computational processes for quality control. We identified and corrected errors, such as misspellings, incorrect georeferences, and mismatched taxonomic names. We also checked for missing or incomplete data, such as absent or vague locality descriptions or missing occurrence dates. We resolved all inconsistencies and missing data by consulting the primary literature, museum specimens, and reference databases at the National Institute of Biodiversity of Ecuador (INABIO) (http://inabio.biodiversidad.gob.ec/) and the Pacific Research Institute of Colombia (https://iiap.org.co/). For additional details regarding computational strategies to avoid duplicate records, please refer to the “Technical validation” section below.

### Data integration

We integrated the data from the individual datasets into a single, unified dataset using the DwC-A common set of terms and standards. According to the informed criterion of the research team, we mapped the individual terms of each dataset to the DwC-A. The mapping process was assisted by the available definition of the Darwin Core terms (https://dwc.tdwg.org/terms/). We used this mapping process to transform the systematized data into the required standardized format.

### Geodatabase

We consolidated a shapefile for the basin that contains layers for vegetation ecosystems and the total number of occurrences, number of species, and number of endemic and threatened species for each ecosystem. This spatial information, including the digital elevation model, was used to generate dynamic three-dimensional shaded relief maps in Rayshader^20^ in R^21^. Endemism is a biogeographical concept, and the construction of the geodatabase provided an opportunity to complement the endemism data recorded in the DwC-A term “establishmentMeans”, which was recorded by the original data operators of the source data files. For this, the species distribution ranges were considered according to the categories of political endemism (i.e. species unique to Colombia or Ecuador) and regional endemism (i.e. species restricted to southern Colombia and northern Ecuador). As a result, we included an additional term (column) in the data tables for plants and terrestrial vertebrates, “Distribution”, that determined the condition of endemism for the recorded species and according to our geographical analysis.

## Data Records

The resulting dataset (i.e. data tables and geodatabase) is available for download at The Knowledge Network for Biocomplexity (KNB) (10.5063/F14F1P6H)^22^, under the Creative Commons Attribution 4.0 International License. The dataset was modified during the manuscript review process; thus, we present the latest version. Although KNB stores previous versions of datasets, these are neither shown nor numbered. Besides the commonly known .csv format, we also compiled the reviewed data tables in the .rds format. The .rds file extension saves a single object that can be interpreted in R^21^ and provides better compression, serialization, and integration within the R environment, when compared to plain .csv files^21^. The available dataset includes three data tables for flora (“m_m_flora_total_2021_ecuador.rds” or “m_m_flora_total_2021_ecuador.csv”, “m_m_flora_voucher_2021_ecuador.rds” or “m_m_flora_voucher_2021_ecuador.csv”, “m_m_flora_observation_2021_ecuador.rds” or “m_m_flora_observation_2021_ecuador.csv”) and one data table for fauna ("m_m_fauna_2021_ecuador.rds” or “m_m_fauna_2021_ecuador.csv”). Two main kinds of records are present in the provided dataset. The first are records based on voucher specimens preserved at herbaria or zoology collections. The second are field observations or records obtained from the literature, without voucher specimens. All literature sources point to its corresponding references in the DwC-A term “References”. Data tables have been encoded in Unicode UTF-8. Additionally, we provide a geodatabase as a shapefile, named “biodiversity_measures_Mira_Mataje.shp” with its accessory files. Because of unavailable coordinates for reported localities, the geodatabase contains 33,027 records, this is 433 fewer records than the total in the presented data tables (33,460 records).

The 33,460 terrestrial records belong to 23,368 flora records and 10,092 fauna records (Fig. 1). The dataset is intended for researchers, policymakers, and stakeholders in biodiversity conservation and management. We encourage users to cite the dataset and acknowledge the original data sources and contributors in their publications and presentations. We also plan to maintain and periodically update the dataset to ensure its relevance and usefulness.

**Fig. 2:**
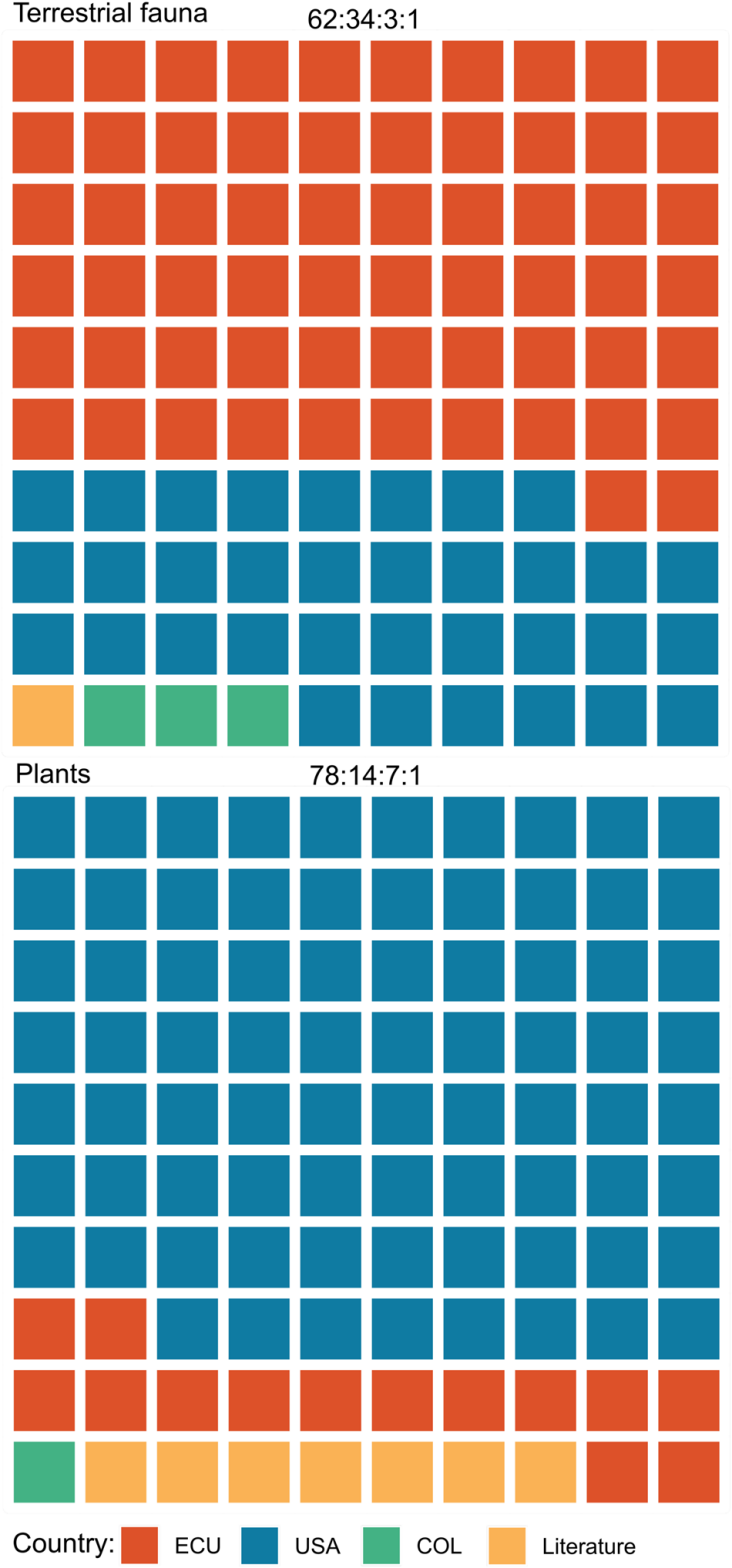
Proportions of contribution by data repositories in different countries for the fauna and flora datasets. Country names follow the ISO 3166 country codes, except for the United Kingdom (UK). Literature sources are also included. Proportions have been rounded to the nearest whole number.

**Fig. 3:**
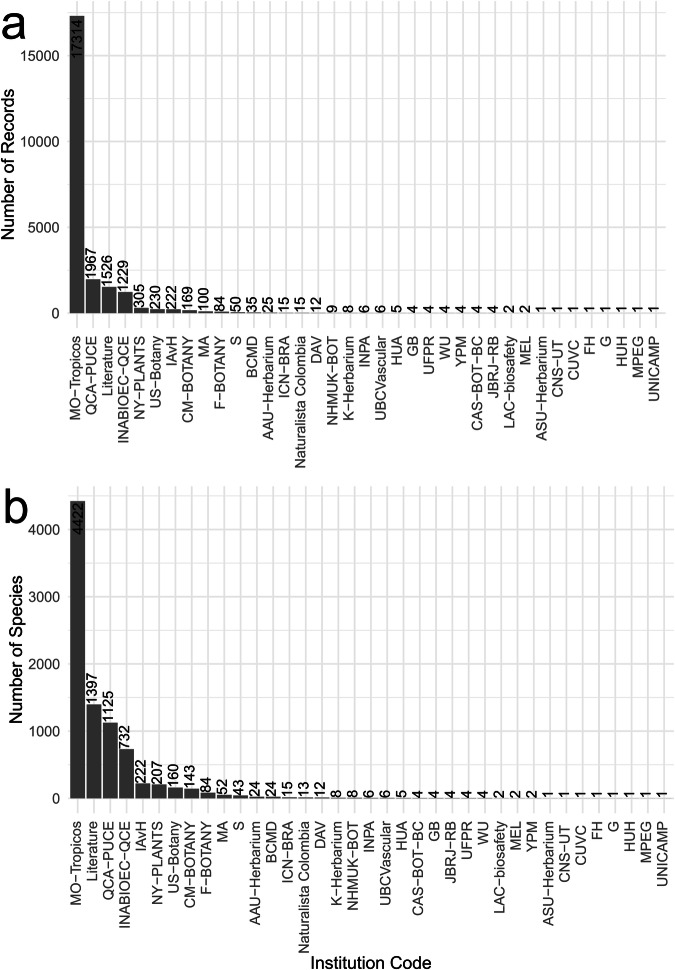
Distribution of flora records (**a**) and flora species (**b**) by repository. The corresponding names for the acronyms are in Supplementary Table S1.

**Fig. 4:**
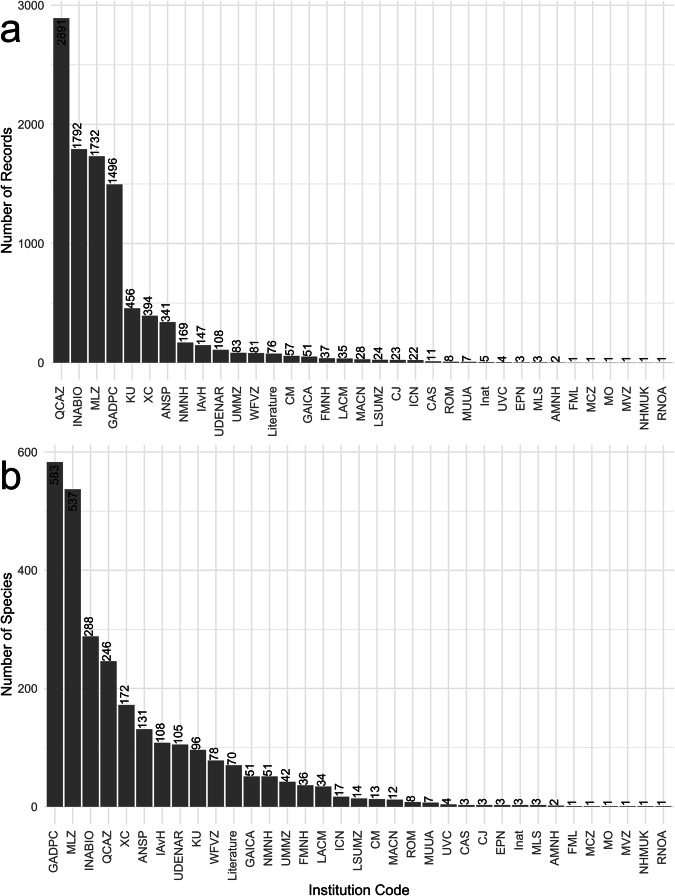
Distribution of fauna records (**a**) and fauna species (**b**) by repository. The corresponding names for the acronyms are in Supplementary Table S2.

The dataset includes data from 13 countries (Fig. 2), with 82.40% of the records from natural history museums and 17.60% from citizen science. For flora, the majority of species and records were obtained from repositories in USA (83.15%), followed by Ecuador (15%) and literature sources (7%) (Fig. 2 and Fig. 3a,b). For fauna, the majority of species and records were obtained from repositories in Ecuador (62%), followed by the USA (34%), and Colombia (3%) (Fig. 2 and Fig. 4a,b). The contrasting differences between the contributions of USA and Ecuador for the flora and fauna datasets, where USA contributes more to flora and Ecuador contributes more to fauna may mirror particular trajectories in the development of biodiversity studies at both countries.

**Fig. 5:**
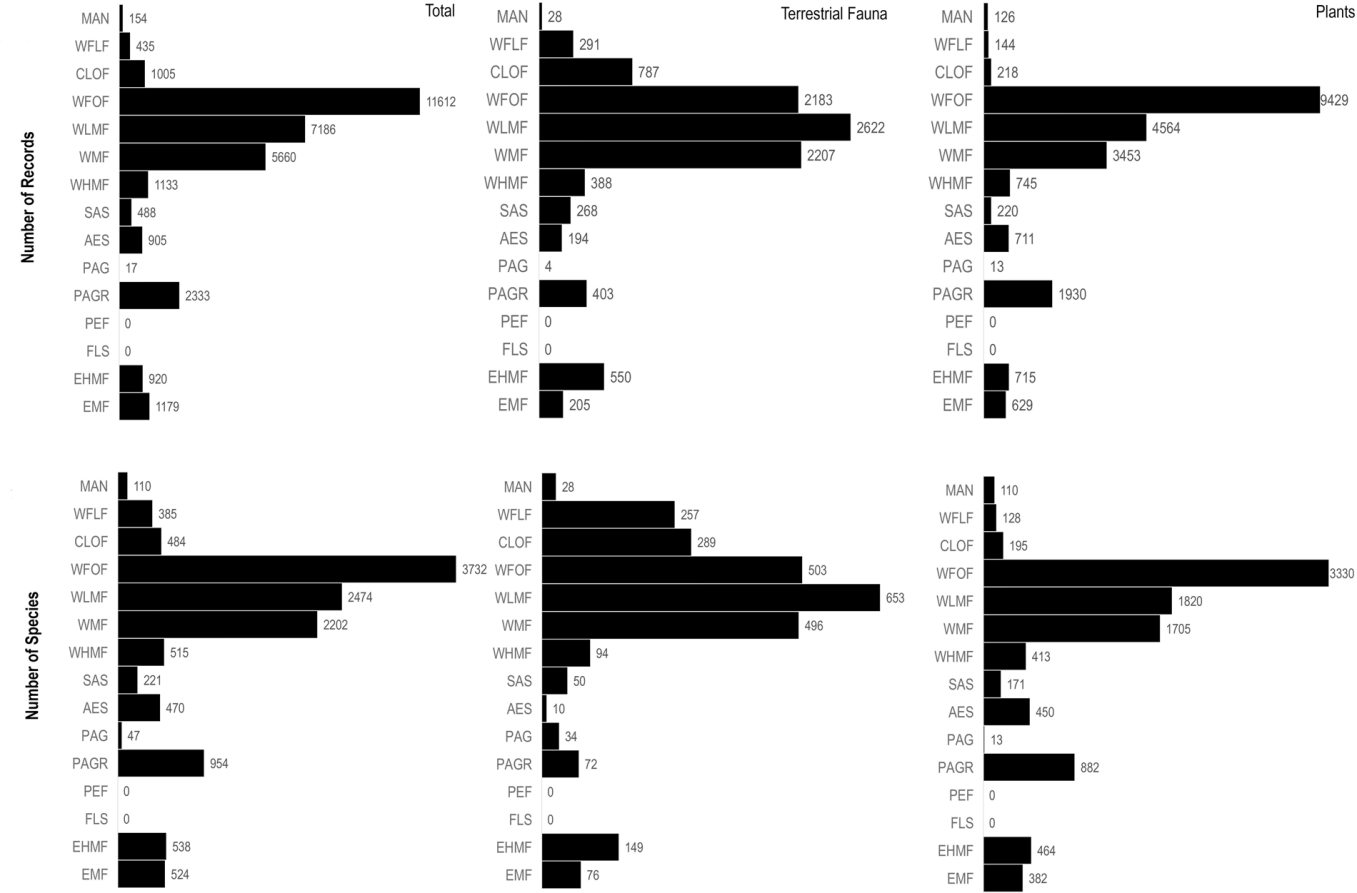
Distribution of records and species in the 15 ecosystems of the Mira-Mataje binational basins. The ecosystems are ordered according to a west-east sequence. MAN= Mangrove, WFLF= Western Flooded forest, CLOF= Chocó Lowland forest, WFOF= Western Foothill forest, WLMF= Western Low montane forest, WMF= Western montane forest, WHMF= Western high montane forest, SAS= Semi-deciduous Andean shrubland, AES= Andean evergreen shrubland, PAG= Páramo grassland, PAGR= Páramo grassland and rose forest, PEF= Páramo evergreen forest, FLS= Flooded shrubland, EHMF= Eastern high montane forest, EMF= Eastern montane forest. Numbers are reported within each ecosystem; thus, totals represent repeated species observations.

**Fig. 6:**
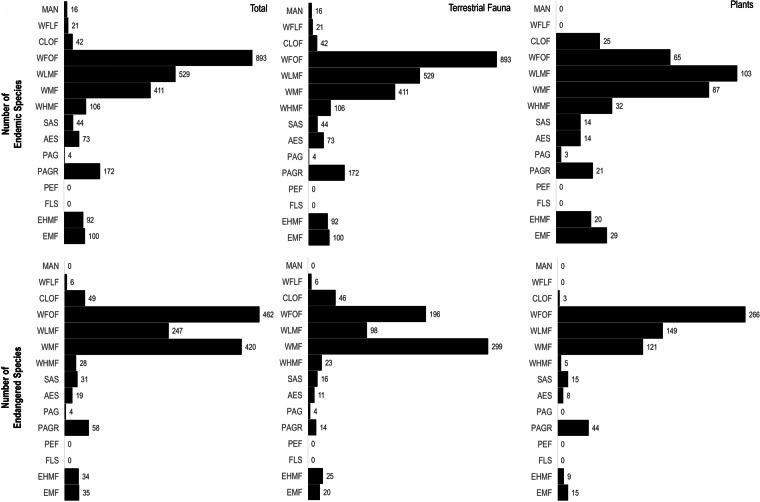
Distribution of endemic and endangered species in the 15 ecosystems of the Mira-Mataje binational basins. The ecosystems are ordered according to a west-east sequence. MAN= Mangrove, WFLF= Western Flooded forest, CLOF= Chocó Lowland forest, WFOF= Western Foothill forest, WLMF= Western Low montane forest, WMF= Western montane forest, WHMF= Western high montane forest, SAS= Semi-deciduous Andean shrubland, AES= Andean evergreen shrubland, PAG= Páramo grassland, PAGR= Páramo grassland and rose forest, PEF= Páramo evergreen forest, FLS= Flooded shrubland, EHMF= Eastern high montane forest, EMF= Eastern montane forest. Numbers are reported within each ecosystem; thus, totals represent repeated species observations.

The data records in both data tables (i.e. “m_m_flora_2021_ecuador.rds” and “m_m_fauna_2021_ecuador.rds”) represent 15 ecosystems in the Mira-Mataje binational basins. The greatest number of records (11,612) came from the Western Foothill Forests (WFOF). Terrestrial fauna records were more abundant in the Western Low Montane Forest (WLMF) ; in contrast, flora records reached higher numbers in the Western Foothill Forests (WFOF) (Fig. 5). We estimated 6,821 species, corresponding to 5,553 plant species and 1,268 terrestrial vertebrate species. According to the DwC-A terms “establishmentMeans” (endemic condition) and “threatStatus-IUCN” (threat status), the dataset has recorded an estimated 540 endemic species and 612 species at risk of extinction (Table 1). The results of our geographical assessment of endemism are described in section “Information on diversity, endemism, and risk of extinction” (Fig. 6).

**Table 1:** Number of units of observation and species of fauna and flora recorded for the Mira-Mataje binational basin.

Groups	Number of records	Number species	Number of endemic species	Number of threatened species
Reptile	693	78	13	27
Mammals	1230	170	11	13
Amphibians	3616	144	35	45
Birds	4553	876	0	210
Plants	23368	5555	481	317
TOTAL	33460	6823	540	612

### Data table for fauna

**Fig. 7:**
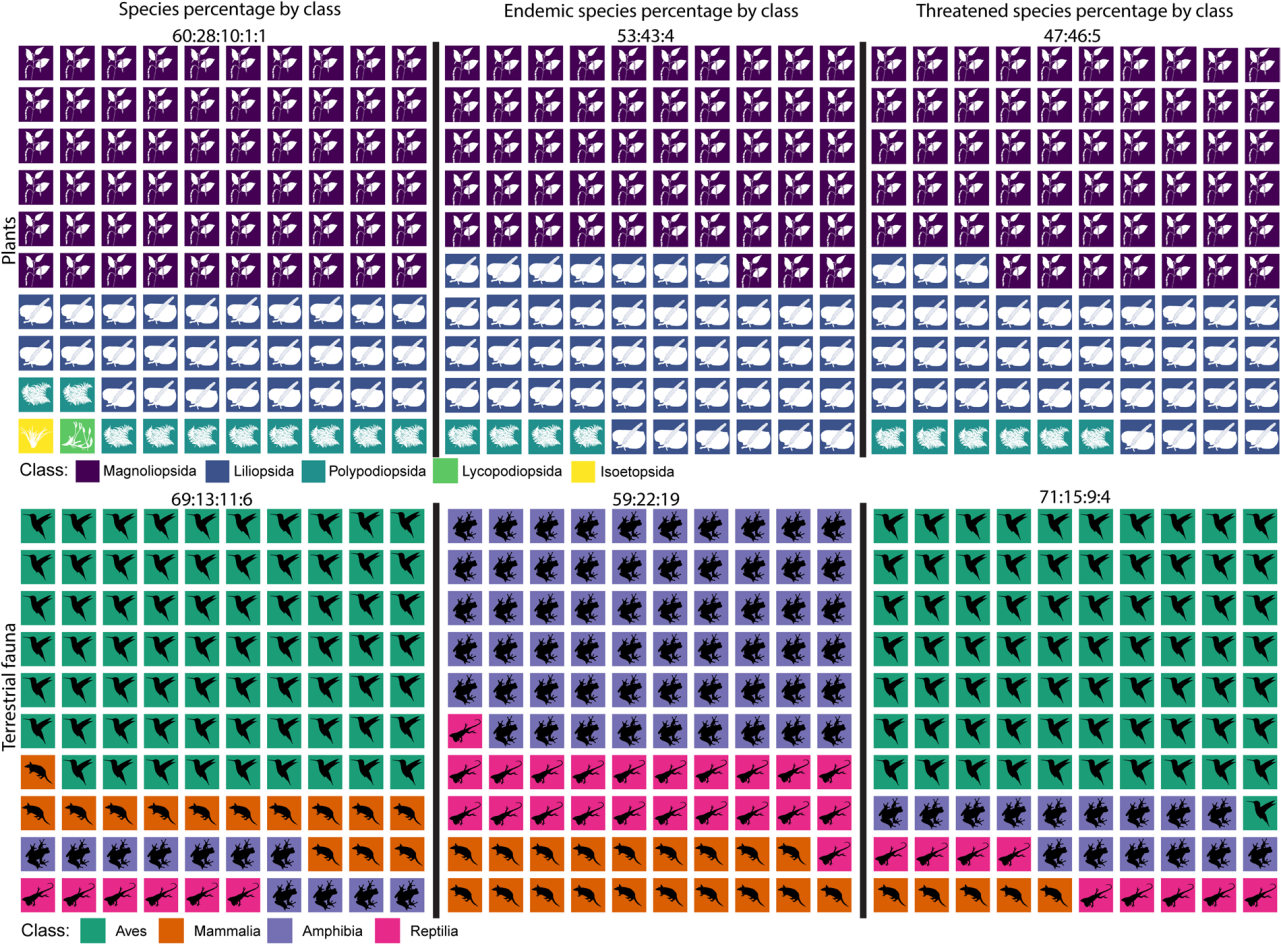
Proportions of the diversity of plants and fauna according to the number of species, endemic species, and threatened species. Proportions have been rounded to the nearest whole number.

In the data table “mira_mataje_fauna_2021_ecuador.rds”, records came from 35 repositories(e.g. museums) in seven countries, with literature as an additional source^23–76^ (Supplementary Table S1). The most relevant repositories, both in the number of records and species, include QCAZ^77–79^, INABIO, MLZ, and GADPC (Fig. 4). The fauna data table corresponds to 1268 species, from which 69.08% are birds, 13.41% mammals, 11.36% amphibians, and 6.15% reptiles (Fig. 7). A total of 670 records have information on endemism, that represent 59 vertebrate species, of which 59.32% are amphibians, 22.03% reptiles, and 18.64% mammals (Fig. 7). A total of 1302 records have information on threatened conservation status, that represent 295 species at risk of extinction; corresponding to 71.19% birds, 15.25% amphibians, 9.15% reptiles, and 4.41% mammals (Fig. 7).

### Data tables for flora

In the data table “mira_mataje_flora_2021_ecuador.rds”, flora records came from 36 repositories, in 12 countries, with literature as an additional source^24,30,74,80–111^ (Supplementary Table S2). The most relevant repositories, both in the number of records and species, include MO-Tropicos, QCA-PUCE^112^, and INABIOEC-QCE, (Fig. 3). Literature sources also provided a representative number of records and species. The flora data table represents 60.18% species of Magnoliopsida, 28.24% of Liliopsida, and 9.95% of Polypodiopsida. A total of 1073 records have information on endemism, from which 481 species are registered as endemic to the southwest of Colombia and northern Ecuador; 52.60% of the species in Magnoliopsida, 43.03% in Liliopsida, and 3.74% in Polypodiopsida are endemic (Fig. 7). A total of 758 records have information on threatened conservation status (vulnerable, endangered, and critical) from which 317 flora species are at risk of extinction, corresponding to 47.31% Magnoliopsida, 46.69% Liliopsida, and 5.36% Polypodiopsida (Fig. 7).

### Information on diversity, endemism, and risk of extinction in the flora and fauna data tables

**Fig. 8:**
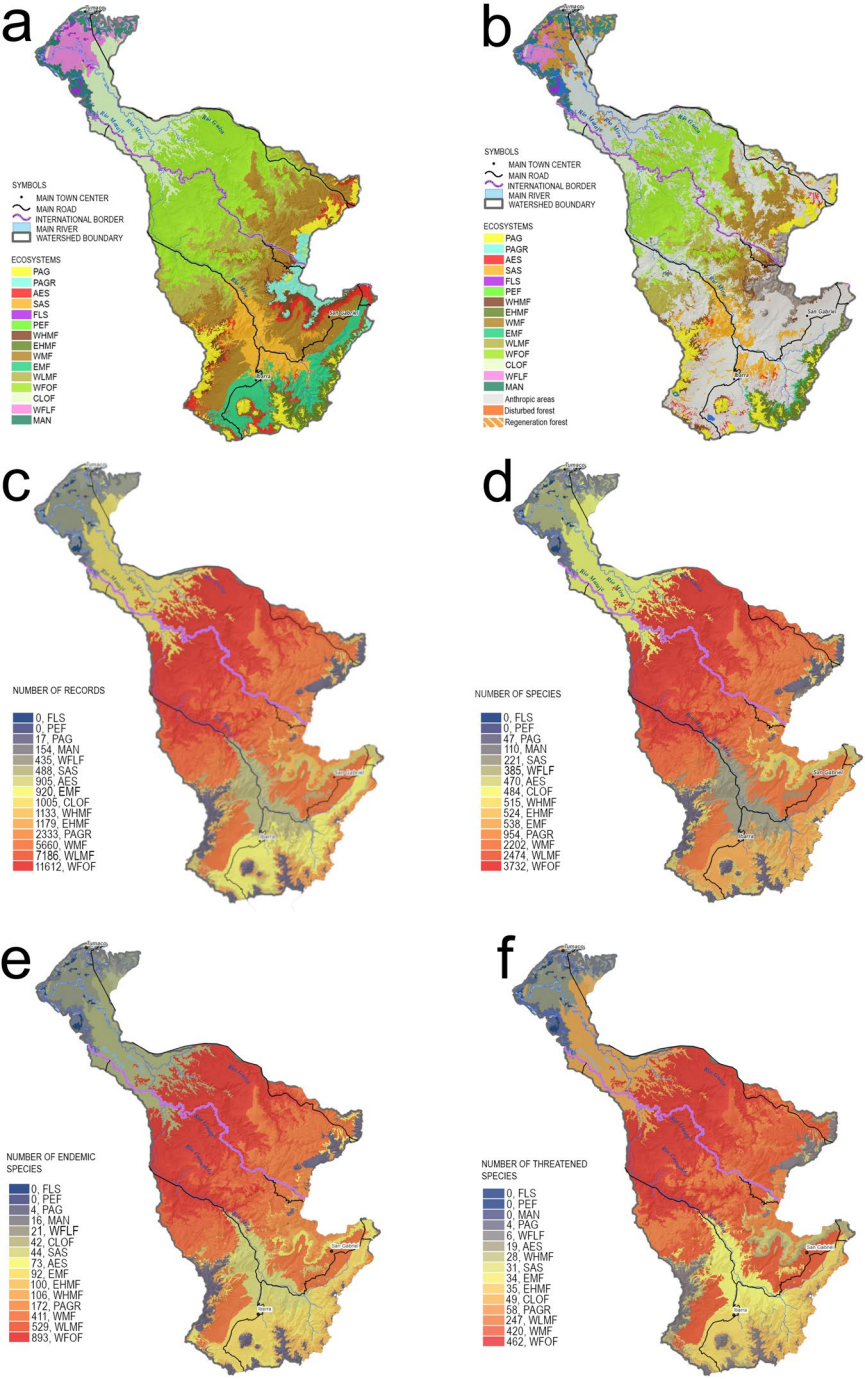
Flora and fauna richness distribution in the Mira-Mataje binational basin. (**a**) Plant ecosystems, (**b**) remnant plant ecosystems, (**c**) number of records by ecosystem, (**d**) number of species, (**e**) number of endemic species, (**f**), number of species. Numbers are reported within each ecosystem; thus, totals represent repeated species observations.

The diversity of flora (5,553 spp.) and fauna (1,268 spp.) of the Mira- Mataje binational basin is equivalent to 10% of the plants and 26% of the vertebrates in the Tropical Andes and Chocó biodiversity hotspots as defined by Myers *et al*.^1^. However, the Mira- Mataje binational basin covers an area of 0.07% of both biodiversity hotspots combined. The ecosystemic mosaic of the Mira-Mataje binational basin influences the distribution of the flora and fauna^11^. It includes the evergreen biomes of the Chocó lowlands (i. e. lower Mira basin) and the low montane forests of the western foothills of the Andes (i.e. middle Mira basin). The altitudinal bands corresponding to the former ecosystems have the highest diversity of the gradient (Fig. 8). The highest number of species (3732) and endangered species (462) occurs in the Western Foothill Forest (WFOF) (Fig. 8). As a complement to Fig. 8, we have made available a set of dynamic 3D shaded-relief map renders in Figshare (10.6084/m9.figshare.23499180.v4)^113^.

Terrestrial fauna diversity is the richest in the Western Low Montane Forest (WLMF), with a total of 653 species and endemic species are more numerous in the Western Foothill Forest, with 893 species (Figs. 5 and 6). In contrast, the number of flora species reaches 3330 in the (WFOF), and 103 endemic species in the WLMF (Figs. 5 and 6). The highest number of endangered species in terrestrial fauna is 299 in the Western Montane Forest (WMF). The highest number of endangered species in plants is 266 in the WFOF (Fig. 6).

## Technical Validation

**Fig. 9:**
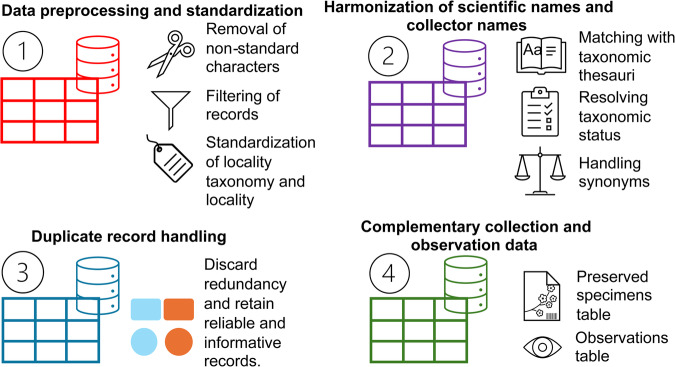
Technical validation process of the presented dataset.

One significant challenge to the presentation of curated biodiversity data sets is the handling of duplicates. Here we define duplicated records as multiple specimens that represent a single object type or collection event^114^. Duplicated records are present at both global online biodiversity databases, like GBIF, and museum repositories, particularly in botanical collections. The same collection event could be found duplicated in various herbaria collections or within a single herbarium. For example, from a total of 58,860 specimens in the Dipterocarpaceae from nine herbaria, 21,075 specimens were selected after discriminating duplicates^115^. Although duplicates, frequently populating the history and constitution of world herbaria, are useful as currency for the interinstitutional exchange of museum material, as a buffer to catastrophic losses, or as foundational material for new herbaria collections^114,116,117^, they also represent problematic data for biodiversity estimates if their condition as duplicates has not been properly recorded, and therefore cannot be discriminated from single data events. Thus, to circumnavigate the issue of duplicates in our data set, we designed a strategy and algorithm in R^21^, and its reference is found in the “Code availability” section, below. The code in R pertains to a data preprocessing and cleaning pipeline designed for botanical and zoological occurrence data. This pipeline comprises several steps aimed at harmonizing taxonomic names, standardizing collector names, identifying and handling duplicate records, and segmenting the dataset for further analysis (Fig. 9). Because duplicates occurred more intensively in the botanical data tables, our focus was on this particular group of data; however, duplicates were also screened in the fauna dataset.

### Data preprocessing and standardization

The raw botanical data underwent a series of preprocessing steps to ensure data quality and standardization. This included the removal of non-standard characters, filtering to include only specimen records, and standardization of taxonomic and locality information for consistency across the dataset. Packages tydyverse^118^ and taxisze^119,120^ where used along the pipeline. The original raw data tables were in .csv format and imported to R. Taxonomic thesauri from The Catalogue of Life (TCF)^121^, World Checklist of Vascular Plants (WCVP)^122^, and World Flora Online (WFO)^123^ were loaded into data frames for taxonomic standardization purposes. By applying regular expressions, unnecessary characters and words were removed or replaced from all data columns which could hinder subsequent taxonomic harmonization and the discrimination of duplicate data. We cleaned, replaced, and standardized specific character patterns in the data tables; such as various representations of null records, whitespace characters or extra spaces, accents, Latin letters, and converted character strings to sentence case. Non-ASCII characters were transformed to ASCII, and unnecessary punctuation marks, separators, and special characters were removed. We placed emphasis on taxonomic and locality information in key DwC-A columns, such as “scientificName”, “recordedBy”, and “locality”.

### Harmonization of scientific and collector names

Scientific names for the plant data set ("scientificName” in the DwC-A terminology) were standardized according to the International Code of Nomenclature for algae, fungi, and plants^124^. All unique 6,808 plant taxa names that were found in our dataset were compared with the WCVP -Version 12^122^. Several scientific names were not found in the WCVP database, so these were compared with the WFO version December 2023^123^, the TCF^121^, and the Tropicos database of the Missouri Botanical Garden (accessed in December 2023, https://tropicos.org), the latter using the API provided by the taxize package^119,120^. Once applied, this approach allowed the identification of multiple synonyms that were updated to accepted scientific names. Besides the DwC-A “scientificName” term, the harmonization also updated the “scientificNameAuthorship”, “family”, “genus”, “specificEpithet”, and “infraspecificEpithet” terms.

To resolve distinct taxonomic determinations across herbaria for duplicates of the same collection event. we applied a prioritization scheme to achieve standardized scientific names. This process was necessary due to potential inconsistencies, synonyms, illegitimate names, misapplied names, and outdated names often present in botanical datasets^115,125^. This prioritization typically followed a hierarchy where accepted names were given the highest priority, followed by synonyms, illegitimate names, misapplied names, and finally invalid names. In some cases, synonyms were updated to their corresponding accepted names based on the WCVP taxonomy. The harmonized scientific names underwent validation and standardization checks to ensure completeness, correctness, and adherence to established nomenclatural rules and conventions. This validation step involved cross-referencing with taxonomic thesauri to confirm the accuracy and validity of the scientific names.

Collector names were parsed and cleaned to extract consistent surnames, ensuring harmonization and standardization across varied collector name formats and irregularities. The the DwC-A term “recordedBy” column was standardized by extracting the collectors’ surnames using regular expressions, and in the few cases where automatic extraction was not possible, the surnames were extracted manually.

### Duplicate records handling

Duplicate records were identified mainly on collector names ("recordedBy” in DwC-A terminology) and record numbers ("recordNumber in DwC-A terminology). When any of both DwC-A terms were available, we used geographic ("decimalLatitude” and “decimalLongitude” in DwC-A terminology) and locality ("locality” in DwC-A terminology) data. Yet, caution and informed decisions are required when using location data, as botanical collectors often tend to apply generalized locality descriptions for collection batches, a practice that obscures the precise identity of recalcitrant duplicate records. Therefore, to obtain a robust flora data table, we filtered out all occurrences that did not have data for the “recordedBy” and “recordNumber” columns, and which were not voucher specimens. This discrimination was performed to ensure the taxonomic reliability of the compiled occurrences. Through this first filtering step, the preliminary flora data table went from 238,219 to 60,094 occurrences.

Duplicate records were prioritized based on the identification method, herbarium information, and taxonomic rank. This prioritization ensured the retention of the most informative and reliable duplicate records while discarding redundant or less informative duplicates. This framework included the following prioritization criteria. 1) Identified by: Records with explicit identification information, such as those identified by experts or through detailed documentation, were prioritized over records without clear identification sources. 2) Herbarium information: Records associated with reputable herbarium institutions, indicated by institution codes, were given higher priority due to the reliability and authenticity typically associated with specimens stored in established herbaria. 3) Taxonomic rank: The taxonomic rank of the specimens, categorized as species, genus, or family, was considered in the prioritization process. Records with higher taxonomic resolution (e.g., species-level identification) were prioritized over records with lower taxonomic resolution (e.g., family-level identification). The prioritization algorithm involved sorting the duplicate records based on the aforementioned criteria in a hierarchical manner. Specifically, records were sorted first by the identification source (identified by or not), then by the associated herbarium (institution code), and finally by the taxonomic rank of the specimen. Duplicate records were evaluated based on the prioritization algorithm, with higher-priority records retained in the dataset. Redundant duplicates, i.e., records that did not offer additional information or value beyond the retained records, were systematically removed from the dataset. False negatives and mismatches were identified for further review and resolution. The final product, after the described approach of filtering and discrimination of records, resulted in a final data table containing 23,368 plant records.

### Voucher specimens and observations

For the plant data set, a complementary data table was generated to encompass occurrences lacking collector names, record numbers, or preservation status, providing a comprehensive overview of both records based on preserved specimens and records based on observations or literature sources (i.e. voucherless records). The DwC-A term “basisOfRecord” provides a controlled vocabulary that differentiates between those records that possess a museum voucher and those that are observations or based on literature sources. For the former, the term “PreservedSpecimen” was used, and for the latter the most common term used was “HumanObservation”. The DwC-A term “basisOfRecord” should be interpreted with the DwC-A term “References” to understand the source of observation records. An additional DwC-A term, “institutionCode”, provides differentiation for those records that belong to institutions and those that belong to literature sources. Both data tables, preserved specimens and observations, provided a comprehensive view of the available botanical occurrence data. These two tables were then merged for the presentation of a global botanical data table. Thus, three tables were deemed necessary, the first with voucher museum specimens, the second with voucherless observations, and the third with merged records from the previous two tables. We present records in this way to facilitate future use and research.

## Usage Notes

To the best of our knowledge, the datasets we have presented for the Mira-Mataje binational basins are unique, as it is the first time a single systematized and consolidated dataset is available for the region. Because of the diverse formats and approaches to tabulating and classifying data in the reviewed sources, a manual validation was necessary to systematize all dispersed data into a single and coherent dataset under the universal and common DwC-A standard. The R statistical programming language^21^ is required to open the provided data tables under the .rds extension, with the advantage of a consistent character codification and a proper analytical environment. The provided comma-separated values files allow universal access to the data. The accompanying geodata is in the common geospatial vector data, which consists of the main file, index file, and database table, and is readable in all common geoinformatics platforms. The open-access release of this Neotropical biodiversity dataset is anticipated to unite stakeholders interested in preserving this highly endangered region.

### Supplementary information

Supplementary Information

## Data Availability

The code for the “Technical validation” section is publicly available at https://github.com/roberto-roman/mira-mataje_duplicates/tree/main. This code requires and works with large taxonomic data tables that have been mentioned in the “Technical Validation” section and that are available for download at The Knowledge Network for Biocomplexity (KNB) (10.5063/F14F1P6H)^22^. The code for generating the 3D shaded-relief maps is publicly available at https://github.com/PJV-Ecu/3D-shaded-relief-maps-using-Rayshader.git.
