# Supplementary material for: The Tropical Andes Biodiversity Hotspot: A Comprehensive Dataset for the Mira-Mataje Binational Basins
Source: Sci Data. 2024 Jul 16;11:782. doi: 10.1038/s41597-024-03463-1 (PMC11252388; doi:10.1038/s41597-024-03463-1)
Supplement: Supplementary file 1 — Supplementary Information [file 41597_2024_3463_MOESM1_ESM.pdf]

Supplementary Information

The Tropical Andes Biodiversity Hotspot: A Comprehensive Dataset for the Mira-Mataje Binational Basins

Table of Contents

Supplementary Table 1..2

Supplementary Table 2..4

Supplementary Table S1

The Tropical Andes Biodiversity Hotspot: A Comprehensive Dataset for the Mira-Mataje Binational Basins

Country	Repository	CODE	Amphibia		Birds		Mammalia		Reptilia	
			Records	Species	Records	Species	Records	Species	Records	Species
ECU	Museo de Zoología, PUCE	QCAZ	1970	92	159	60	314	51	448	44
ECU	Instituto Nacional de Biodiversidad, Ecuador	INABIO	980	79	169	128	610	69	33	13
USA	Moore Laboratory of Zoology	MLZ			1732	537				
ECU	Gobierno autónomo descentralizado de la provincia del Carchi, Ecuador	GADPC	58	34	1262	473	157	63	19	13
USA	Museum of Kansas University	KU	357	42	53	44			46	10
USA	Xeno-Canto	XC			394	172				
USA	Academy of Natural Sciences, Philadelphia	ANSP			341	131				
USA	Smithsonian Institution (USNM)	NMNH	93	32	2	2	5	4	69	16
COL	Instituto de Investigación de Recursos Biológicos Alexander von Humboldt	IAvH	60	30	77	69	3	3	7	7
COL	Universidad de Nariño	UDENAR			78	78	30	27		
USA	University of Michigan, Museum of Zoology	UMMZ	7	2	31	27	45	13		
USA	Western Foundation of Vertebrate Zoology	WFVZ			81	78				
NA	Literature	Lit	20	16	28	28	8	8	20	1 8
COL	Proyecto GAICA	GAICA			51	51				10
USA	Carnegie Museum	CM	29	2	2	2	26	10		
USA	The Field Museum of Natural History	FMNH			25	25	12	11		
USA	Los Angeles County Museum	LACM			29	29	6	5		
ARG	Museo Argentino de Ciencias Naturales "Bernardino Rivadavia"	MACN					28	12		
USA	Louisiana State University, Museum of Zoology	LSUMZ			24	14				
ECU	Centro Jambatu	CJ	23	3						
COL	Instituto de ciencias naturales, Universidad de Colombia	ICN					7	7	15	10
USA	California Academy of Sciences	CAS	11	3						

Country	Repository	CODE	Amphibia		Birds		Mammalia		Reptilia	
			Records	Species	Records	Species	Records	Species	Records	Species
CAN	The Royal Ontario Museum	ROM			8	8				
COL	Asociación GAICA	GAICA			51	51				2
COL	Universidad de Antioquia (UdeA)	MUUA	5	5					2	2
USA	iNaturalist	Inat					3	1	2	2
COL	Universidad del Valle del Cauca	UVC			4	4				
COL	Universidad de La Salle	MLS	1	1					2	2
ECU	Escuela Politéncica Nacional	EPN					1	1	2	1
USA	America Museum of Natural History	AMNH	1	1					1	1
ARG	Fundación Miguel Lillo	FML							1	1
COL	Red Nacional de Observadores de Aves, Colombia	RNOA			1	1				
LIT	Modern art museum in Vilnius	MO	1	1						
UK	Natural History Museum, London, United Kingdom	NHMUK					1	1		
USA	Museum of Comparative Zoology, Harvard University	MCZ	1	1						
USA	Museum of Vertebrate Zoology, University of California, Berkeley	MVZ			1	1				

Biodiversity repositories that were used as sources for data systematization and the corresponding number of records and species of fauna found at the Mira-Mataje Binational Basin.

Supplementary Table S2

The Tropical Andes Biodiversity Hotspot: A Comprehensive Dataset for the Mira-Mataje Binational Basins

MO

Country	Repository	CODE	Records	Species
USA	Missouri Botanical Garden	MO-TROPICOS	17314	4022
ECU	Herbario de la Universidad Catolica del Ecuador	QCA-PUCE	1967	1125
LIT	Literature	Literature	1526	1397
ECU	Herbario Nacional del Ecuador (Instituno Nacional de Biodiversidad)	INABIOEC-QCE	1229	732
USA	New York Botanical Garden	NY-PLANTS	305	207
USA	United States National Herbarium - Smithsonian	US-Botany	230	160
COL	Instituto Alexander von Humboldt	IAvH	222	222
USA	Herbarium of the Carnegie Museum of Natural History	CM-BOTANY	169	267
ESP	CSIC-Real Jardín Botánico	MA	100	52
USA	Field Museum of Natural History (Botany), Seed Plant Collection	F-BOTANY	84	84
SUE	Swedish Museum of Natural History, Phanerogamic Botanical Collections	S	50	43
USA	Bioversity Collecting Mission Database	BCMD	35	24
DIN	Herbarium of the University of Aarhus	AAU-Herbarium	25	24
BRA	Herbário do Instituto de Ciências Naturais	ICN-BRA	15	15
COL	Naturalista Colombia	Naturalista Colombia	15	13
USA	University of California, Davis Herbarium	DAV	12	12
UK	Natural History Museum (London), Collection Specimens	NHMUK-BOT	9	8
UK	Royal Botanic Gardens, Kew	K-Herbarium	8	8
CAN	University of British Columbia Herbarium, Vascular Plant Collection	UBCVascular	6	6

Country	Repository	CODE	Records	Species
BRA	Instituto Nacional de Pesquisas da Amazônia (INPA)	INPA	6	6
COL	Herbario de la Universidad de Antioquia (UdeA)	HUA	5	5
USA	California Academy of Sciences, San Francisco	CAS-BOT-BC	4	4
SUE	University of Gothenburg, Gothenburg Herbarium	GB	4	4
BRA	Rio de Janeiro Botanical Garden Herbarium Collection	JBRJ-RB	4	4
BRA	Herbário do Departamento de Botânica, da Universidade Federal do Paraná	UFPR	4	4
AUT	Institute of Botany, University of Vienna	WU	4	4
USA	Yale Peabody Museum of Natural History	YPM	4	2
COL	LAC-biosafety	LAC-biosafety	2	2
AUS	National Herbarium of Victoria	MEL	2	2
USA	Arizona State University Herbarium	ASU-Herbarium	1	1
USA	Plant Resources Center, University of Texas at Austin	CNS-UT	1	1
COL	Colección de referencia del Herbario CUVC de la Universidad del Valle	CUVC	1	1
USA	Harvard University-Farlow, Herbarium	FH	1	1
SUI	Conservatoire et Jardin botaniques de la Ville de Genève	G	1	1
USA	Harvard University Herbaria: Vascular Plants	HUH	1	1
BRA	Museu Paraense Emílio Goeldi	MPEG	1	1
BRA	Universidad Estatal de Campinas	UNICAMP	1	1

Biodiversity repositories that were used as sources for data systematization and the corresponding number of records and species of flora found at the Mira-Mataje Binational Basin.
